# Imaging cellulose synthase motility during primary cell wall synthesis in the grass *Brachypodium distachyon*

**DOI:** 10.1038/s41598-017-14988-4

**Published:** 2017-11-08

**Authors:** Derui Liu, Nina Zehfroosh, Brandon L. Hancock, Kevin Hines, Wenjuan Fang, Maria Kilfoil, Erik Learned-Miller, Karen A. Sanguinet, Lori S. Goldner, Tobias I. Baskin

**Affiliations:** 1 Physics Department, University of Massachusetts, Amherst, MA 01003 USA; 2 Biology Department, University of Massachusetts, Amherst, MA 01003 USA; 3 Computer Science, University of Massachusetts, Amherst, MA 01003 USA; 4 Present Address: Department of Plant Systems Biology, VIB, 9052 Gent Belgium; 5 Present Address: Department of Crop and Soil Sciences, Washington State University, Pullman, WA 99164 USA

## Abstract

The mechanism of cellulose synthesis has been studied by characterizing the motility of cellulose synthase complexes tagged with a fluorescent protein; however, this approach has been used exclusively on the hypocotyl of *Arabidopsis thaliana*. Here we characterize cellulose synthase motility in the model grass, *Brachypodium distachyon*. We generated lines in which *mEGFP* is fused N-terminal to *BdCESA3* or *BdCESA6* and which grew indistinguishably from the wild type (Bd21-3) and had dense fluorescent puncta at or near the plasma membrane. Measured with a particle tracking algorithm, the average speed of GFP-BdCESA3 particles in the mesocotyl was 164 ± 78 nm min^−1^ (error gives standard deviation [SD], n = 1451 particles). Mean speed in the root appeared similar. For comparison, average speed in the *A. thaliana* hypocotyl expressing GFP-AtCESA6 was 184 ± 86 nm min^−1^ (n = 2755). For *B. distachyon*, we quantified root diameter and elongation rate in response to inhibitors of cellulose (dichlorobenylnitrile; DCB), microtubules (oryzalin), or actin (latrunculin B). Neither oryzalin nor latrunculin affected the speed of CESA complexes; whereas, DCB reduced average speed by about 50% in *B. distachyon* and by about 35% in *A. thaliana*. Evidently, between these species, CESA motility is well conserved.

## Introduction

Cellulose is successful, having a tensile strength rivaling steel and being perhaps the most abundant organic polymer on the planet. Cellulose is synthesized by cellulose synthase, a plasma-membrane enzyme complex that catalyzes the addition of glucose onto the non-reducing end of a 1,4-ß-glucan^1–3^. Within the complex, a catalytic subunit binds UDP-glucose at a cytosolic site and extrudes the glucan extracellularly. The complex comprises multiple subunits and is thought to synthesize concomitantly at least 18 glucans, and possibly as many as 36. Soon after synthesis, the glucans associate laterally, forming a crystalline array, dense with hydrogen bonds, known as a *microfibril*. Large and stiff, the nascent microfibril rapidly becomes enmeshed within the extant cell wall. With the bulk of the microfibril thus immobilized, the continued polymerization of the glucans evidently pushes the synthesizing complex through the plasma membrane, using energy released through hydrolyzing UDP-glucose and possibly also through crystallization.

Besides being successful, cellulose is also enigmatic, particularly with respect to how it is synthesized. Enigmas surround cellulose synthesis in part because the responsible enzymes, when isolated and purified from the plant, are usually inactive. Inactivity, while common, is not universal: fractions from the plasma membrane have been isolated that synthesize cellulose^4,5^. Nevertheless, the preparative method is difficult, yields are relatively low, and for reasons that remain obscure the protocol fails when applied to *Arabidopsis thaliana* ^6^. Inactivity of cellulose synthase isolated from *A. thaliana* is unfortunate because of the powerful molecular and genetic tools available for this species.

Despite frustrating the biochemists, *A. thaliana* has supported a valuable alternative approach. In this approach, pioneered by Paredez *et al*.^7^, a fluorescent-protein label is added to one of the cellulose synthase’s catalytic subunits (named CESA). Through fluorescence microscopy, the movement of the tagged complex can be observed. Because the movement is tied closely to glucan extension, the speed of movement provides a proxy for the reaction rate and thereby can reveal details of the reaction mechanism.

This approach has been used to characterize cellulose synthesis in the hypocotyl of *A. thaliana* seedlings^8,9^. There, the speed of movement is approximately 300 nm min^−1^, a rate that corresponds to each glucan in the complex being elongated by about ten glucose units per second^7^. Imaging tagged CESA complexes has helped show how cellulose synthesis is influenced by the cytoskeleton as well as by specific, regulatory proteins^1–3^.

While the information thus mined has been revelatory, so far the movement of the cellulose synthase complex has to our knowledge been reported for *A. thaliana* only, and in that species almost exclusively for the hypocotyl. It is not known to what extent the results from the *A. thaliana* hypocotyl can be generalized even to other organs, let alone to other species.

To characterize the motility of cellulose synthase in another species, we chose the grass *Brachypodium distachyon*. In view of its small stature and genome, as well as its phylogenetic affinity to wheat and related temperate grasses, *B. distachyon* is emerging as a popular model grass^10,11^. Grasses evolved a cell wall that differs in composition and structure from most other taxa^11,12^, and it is reasonable to allow that the mechanistic details of cellulose synthesis might likewise differ.

Cellulose synthase catalytic subunits are encoded by the *CESA* gene family. In any cell, active cellulose synthase is believed to involve CESA subunits from three distinct clades. For primary cell walls in *A. thaliana*, the clades are named CESA1, CESA3, and CESA6-like. In *B. distachyon*, the CESA family structure has been characterized^13^. It resembles that of other land plants and the putative orthologs in *B. distachyon* have been named to follow as much as possible the nomenclature established for *A. thaliana*. Both species have a small family of *CESA6*-like genes (three in *B. distachyon*; four in *A. thaliana*) and a single *CESA1* gene. In contrast, *A. thaliana* has one *CESA3* gene while *B. distachyon* has two (*BdCESA3* and *BdCESA2*); however, mRNA from *BdCESA2* is weakly expressed in stems, leaves, and roots^13^.

Here, we generated plants in which *B. distachyon* CESA3 and CESA6 are tagged with a green fluorescent protein (GFP), and used them to characterize CESA motility in both root and mesocotyl and as affected by selected inhibitors.

## Results

### Transgenic lines expressing GFP-tagged CESA proteins

**Figure 1:**
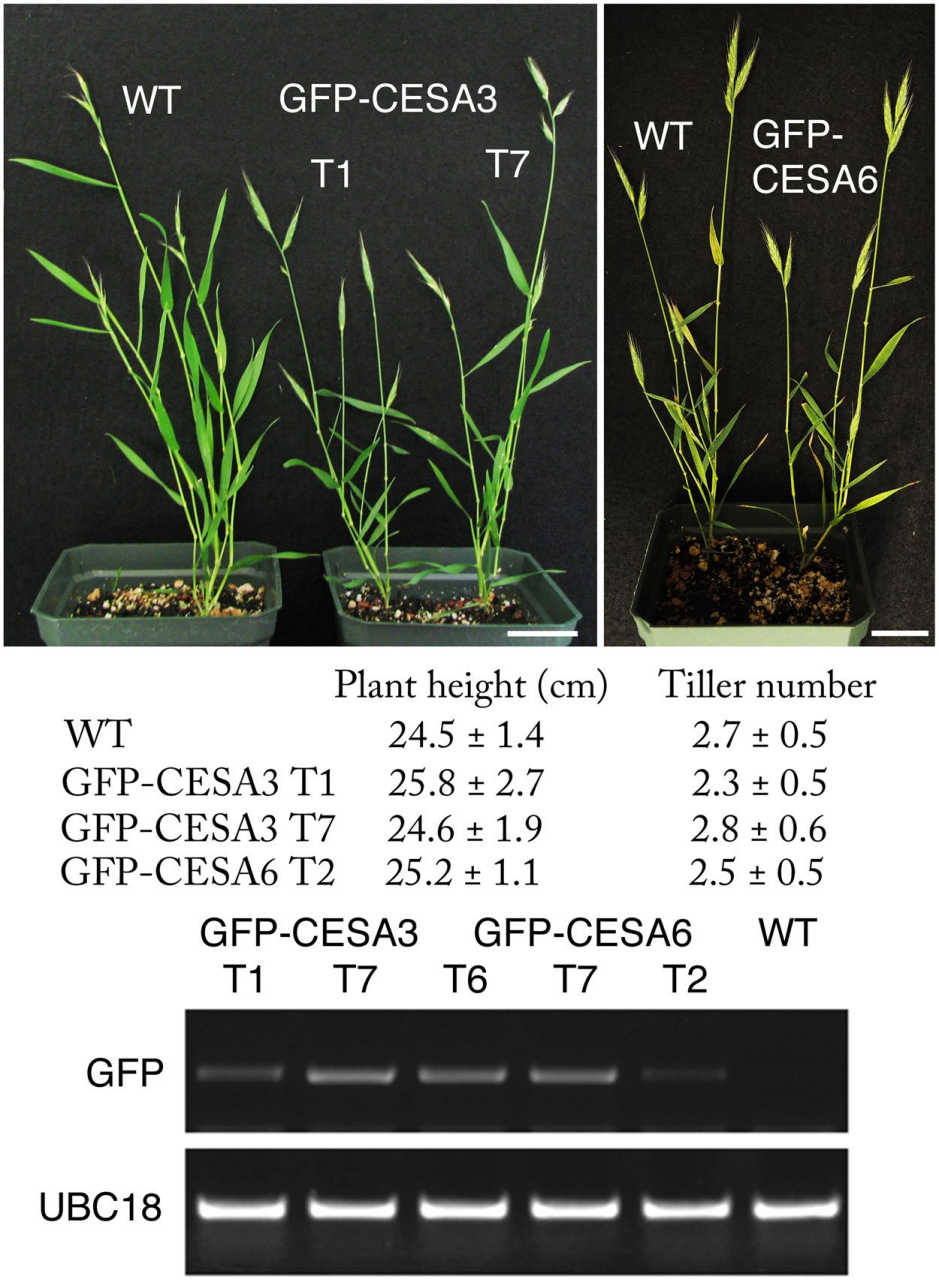
*B. distachyon* transgenic lines expressing GFP-BdCESA proteins. (**Top**) Representative plants at the heading stage grown in the greenhouse. T1 and T7 indicate independent transgenic lines. For GFP-CESA6, line T6 is shown. Scale bars = 3 cm. (**Middle**) Growth parameters for plants at the stage shown above. Data are mean ± SD for 8 to 10 plants from a single experiment. (**Bottom**) RT-PCR detection of mRNA from the transgenes. The first two lanes (T1, T7) are the two GFP-CESA3 lines. UBC18 stands for ubiquitin-conjugating enzyme 18.

To generate plants for imaging CESAs, we made constructs in which *mEGFP* ^14^ is fused to N-terminal *B. distachyon CESA* coding sequences, obtained from wild-type cDNA, and is driven by a *Zea mays* ubiquitin promoter. We worked with *BdCESA3* (putative ortholog of *AtCESA3* ^13^) and *BdCESA6* (one of three *CESA6-like* family members in *B. distachyon* ^13^), in view of their strong expression in seedlings^13^ and the functionality of the *A. thaliana* orthologs when similarly tagged^7,15^. By the second generation after transformation, some lines expressing either construct appeared indistinguishable from the wild type (Fig. 1). Although expression of the transgene varied between the lines, only those with substantially higher expression were associated with aberrant plant development. For imaging, we selected lines with intermediate expression: two lines (T1 and T7) for *GFP-BdCESA3* and one line (T2) for *GFP-BdCESA6*. Qualitatively, these three lines were similar and here we present results for *GFP-BdCESA3*.

### Cellulose synthase complex movement: qualitative observations

**Figure 2:**
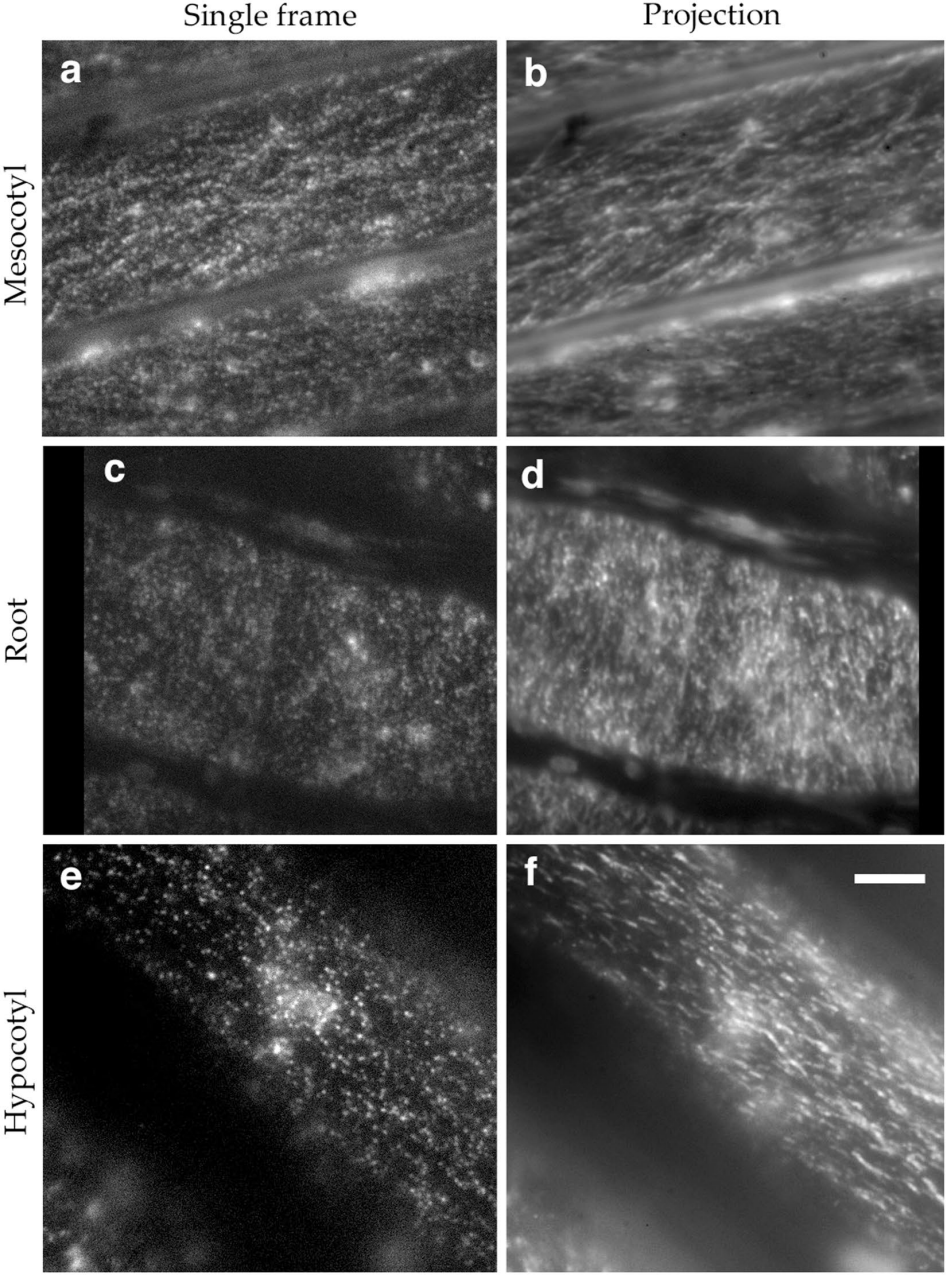
CESA localization and motility. (**a,b**) GFP-BdCESA3 imaged in the *B. distachyon* mesocotyl. (**c,d)** GFP-BdCESA3 imaged in the *B. distachyon* root. (**e,f**) GFP-AtCESA6 imaged in the *A. thaliana* hypocotyl. Panels b, d, & f are average projections of sequences acquired over 4 min, comprising 150 (**b**), 122 (**d**), and 80 (**f**) frames. Bar = 5 µm (all panels are at the same magnification).

In grasses, the first stem internode, made in the embryo, is called the mesocotyl. It is functionally analogous to the hypocotyl and likewise convenient for imaging. For imaging, we used a microscope set up for total internal reflection fluorescence (TIRF) microscopy but illuminated the sample at a sub-critical angle. This approach, sometimes called variable-angle epi-fluorescence microscopy^16^, allows deeper penetration of excitation light into the sample than does TIRF while still reducing background. In the mesocotyl of the tagged lines, bright puncta were present within a narrow focal plane, presumably corresponding to the plasma membrane and a thin layer of cortical cytoplasm (Fig. 2a). Puncta often appeared to be organized in lines and, among puncta, fluorescence intensity varied, even within a single cell, indicating that the endogenous and introduced CESA proteins might compete for space in the complex. In image sequences, particles appeared to move bi-directionally (Movie S1). As a way to assess motility visually, we made average projections, in which a moving particle appears as a line. Although some particles appeared stationary, a large majority of them moved (Fig. 2b).

To extend these results, we imaged tagged cellulose synthase complexes in the root. Helpfully for sub-critical angle illumination, the outer epidermal wall of the root is thinner than that of the mesocotyl but, unhelpfully, *B. distachyon* roots sprout a luxuriant mane of root hairs, making it difficult to bring the root close to the coverslip. Therefore, we exposed roots for several days to silver nitrate, at a concentration that inhibits root hair growth but has little or no effect on the growth of the root itself. In the tagged lines, puncta were visible in the root, and their appearance and motility appeared similar to the mesocotyl (Fig. 2c,d; Movie S2).

Because our intention is to compare CESA motility in *B. distachyon* to that of *A. thaliana*, we obtained a GFP-AtCESA6 line^15^, and imaged the tagged CESA in the hypocotyl. As expected, the GFP-AtCESA6 fluorescence formed puncta at the plasma membrane. In single frames and projections (Fig. 2e,f; Movie S3), the puncta appeared qualitatively similar to those in the *B. distachyon* mesocotyl.

**Figure 3:**
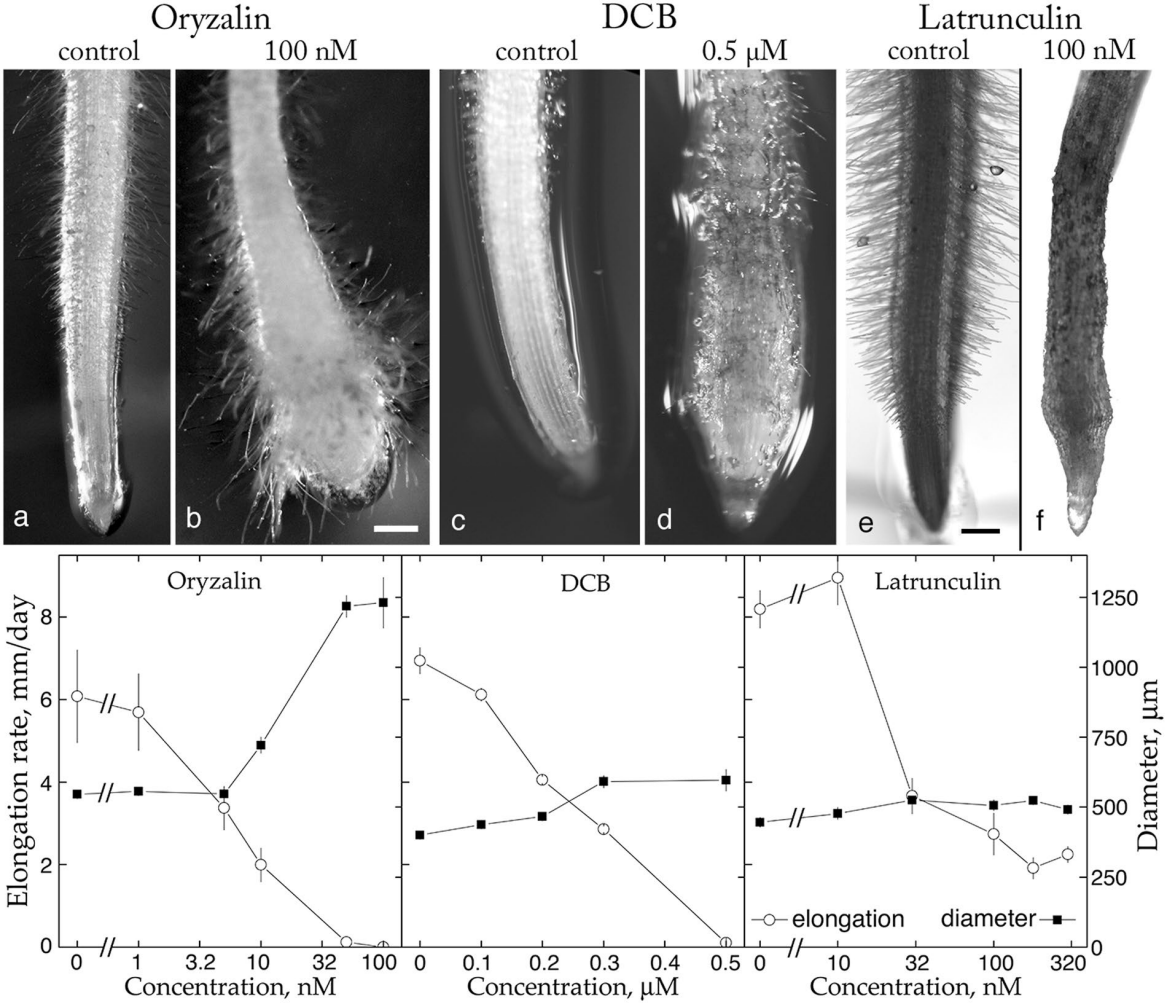
Effects of selected inhibitors on morphology and growth of *B. distachyon* roots. Top: Bright-field images with control root on the left and inhibitor-treated on the right. (**a,b**) 100 nM oryzalin for 48 h; (**c,d**) 0.5 µM DCB for 48 h; and (**e,f**) 250 nM latrunculin for 19 h. Scale bars = 0.3 mm (bar in **b** is for **a** and **b**; bar in **e** is for **c**–**f**). Bottom: Dose-response curves. Symbols plot mean ± SE for a representative experiment with 8 to 12 seedlings per datum. Open circles plot elongation, closed squares plot diameter.

Disruption of cellulose synthesis is indicated when the growth of a cylindrical organ becomes less anisotropic, that is, when the organ swells. Such swelling is caused by several classes of inhibitors, which have been used productively to probe cellulose synthesis. We selected an inhibitor of cortical microtubules, oryzalin; of cellulose synthesis, dichlorbenzyl-nitrile (DCB); and of actin filaments, latrunculin B. First, we ran dose-response curves to determine the sensitivity and responsiveness of *B. distachyon* (Fig. 3). The three compounds inhibited elongation and stimulated radial expansion. Latrunculin also inhibited growth of root hairs. Compared to *A. thaliana*, the grass *B. distachyon* was at least ten times more sensitive to oryzalin, about equally sensitive to DCB, and about five times less sensitive to latrunculin^17,18^. As for responsiveness, in *A. thaliana*, saturating doses of either oryzalin or DCB caused root diameter to triple^17^; whereas in *B. distachyon*, while oryzalin caused almost the same degree of swelling, DCB did not quite double root diameter (Fig. 3). Saturating latrunculin increased root diameter by around 15%, a modest response level that is nevertheless similar in both species.

**Figure 4:**
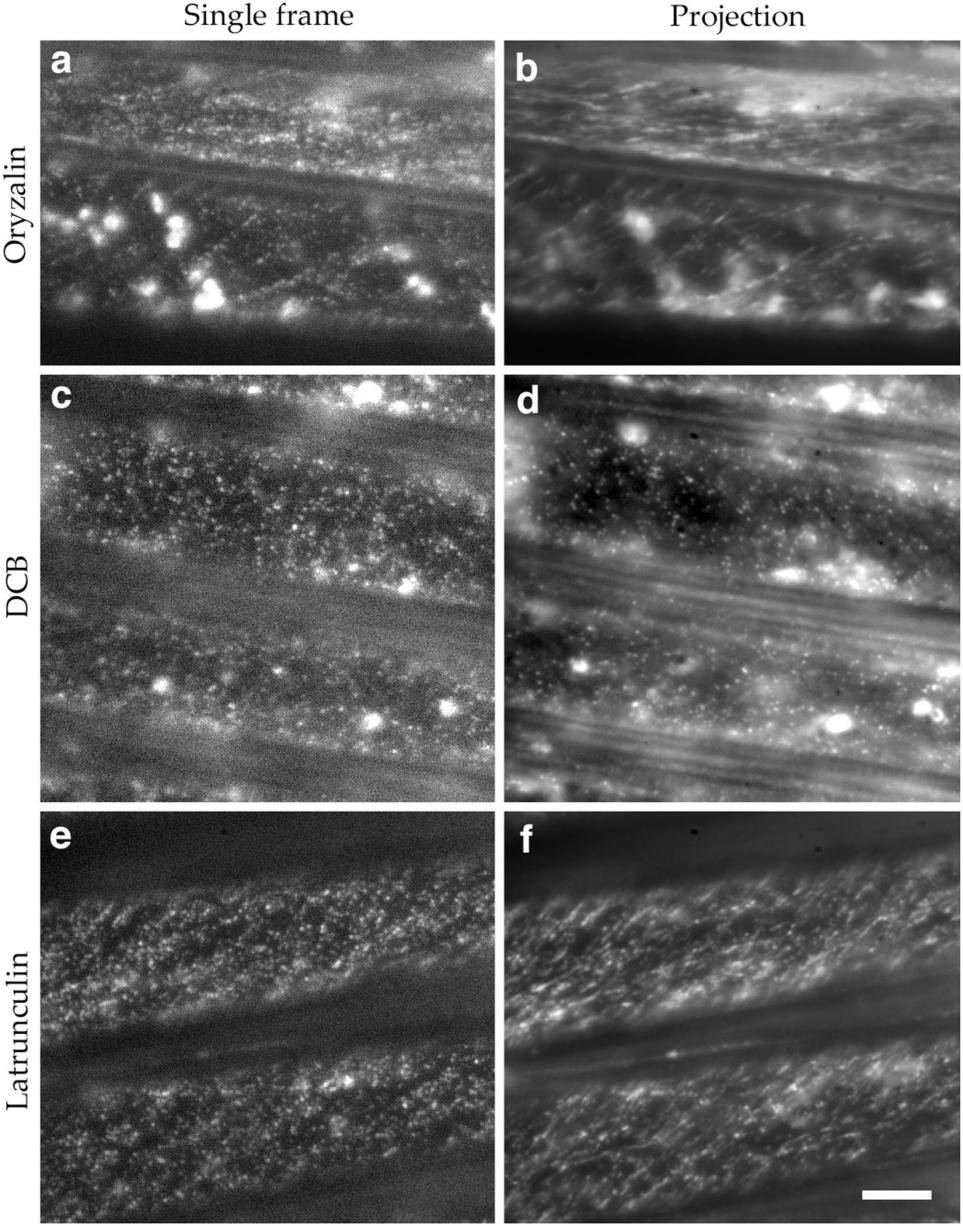
Effect of selected inhibitors on CESA localization and motility in *B. distachyon* mesocotyl cells. (**a,b**) 30 nM oryzalin for 30 min. (**c,d**) 5 µM DCB for 150 min. (**e,f**) 250 nM latrunculin for 60 min. Exposure time was 500 ms. b, d, and f are average projections of 150 frames acquired over 4 min. Imaged plants expressed GFP-BdCESA3. Bar = 5 µm (all panels are at the same magnification).

Cellulose synthase complexes move under the influence of cortical microtubules^7,19^ but the nature of the relation between these elements is not fully understood. In *A. thaliana*, depolymerizing microtubules with oryzalin does not appreciably alter tagged CESA speed, at least for treatment times less than a day^7,20,21^. We treated seedlings for up to 2.5 h with 30 nM oryzalin and observed CESA motility in the *B. distachyon* mesocotyl. Qualitatively, the puncta moved as they did in untreated plants (Fig. 4a,b; Movie S4). After 1 day of treatment at this concentration, the mesocotyls were swollen, indicating activity of treatment.

In *A. thaliana*, the inhibitor of cellulose synthesis, DCB, has been observed to halt CESA motility^22^. Therefore, we treated *B. distachyon* with DCB and examined CESA motility in the mesocotyl. In seedlings treated with 5 µM DCB for 150 min, the density of puncta was not manifestly changed, but, as seen by the limited formation of tracks in the projection image, their motility was curtailed (Fig. 4c,d; Movie S5).

While microtubules have been the focus of efforts expended to understand the control of cellulose synthase motility, when actin is inhibited, roots and stems swell radially^17^, which could indicate a lowered rate of cellulose synthesis. Indeed, actin filaments have been implicated in trafficking CESA complexes^23,24^, and cell walls of seedlings treated with latrunculin have reduced amounts of cellulose^25^. Because short-term treatments with latrunculin did not appear to inhibit cytoplasmic streaming in *B. distachyon*, we exposed seedlings on plates to 250 nM latrunculin for one day prior to imaging. While cytoplasmic streaming was inhibited with that treatment, CESA motility appeared similar to that of controls (Fig. 4e,f; Movie S6).

### Cellulose synthase complex movement: quantification

**Figure 5:**
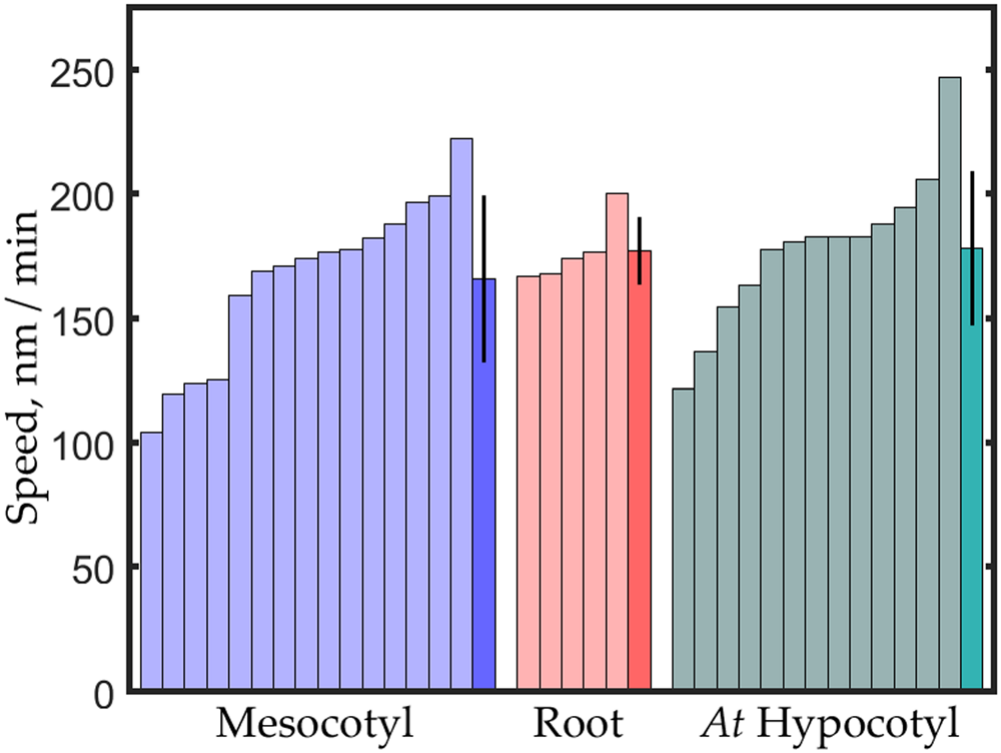
Quantification of CESA motility. Mesocotyl and Root are *B. distachyon*, *At* Hypocotyl is *A. thaliana*. For each treatment, lightly shaded bars plot average speed for tracked particles in a sequence. Darker bars plot mean ± SD of the average speeds shown. Bars are arranged from smallest to largest for clarity. Tracked particle number in each sequence ranged from 26 to 185 (mesocotyl); 49 to 279 (root); 28 to 535 (hypocotyl). For mesocotyl, seedlings were imaged on three occasions with two to four plants each. In separate experiments, roots were imaged on three occasions, with one or two plants each. Hypocotyls were imaged on four occasions with one or two plants each.

To quantify motility, we used a particle-tracking algorithm^26^. This routine finds particles meeting selection criteria and follows them through subsequent frames, without requiring manual intervention. In each sequence, particles that were tracked for longer than 30 seconds were used to determine average speed. In Figs 5 and 7, each bar shows the mean speed of particles in a given image sequence, with the last bar of a treatment group (drawn in a darker shade) showing the mean speed averaged over the sequences in the group.

For the mesocotyl, variability between image sequences (between different cells, often on different plants) was appreciable (Fig. 5). The data include results from both 3- and 4-day-old seedlings, but age did not appear to account for the variability. For the root, average speeds tended to be a little higher than for the mesocotyl but the trend was unlikely to be significant. In view of the need to inhibit root hairs, additional root sequences were not obtained. To allow direct comparisons, we also quantified the tagged CESA motility in the *A. thaliana* hypocotyl (Fig. 5). Again, the variability between cells was notable but comparable to that of *B. distachyon*. Likewise, the mean of the sequences for each species appeared similar.

**Figure 6:**
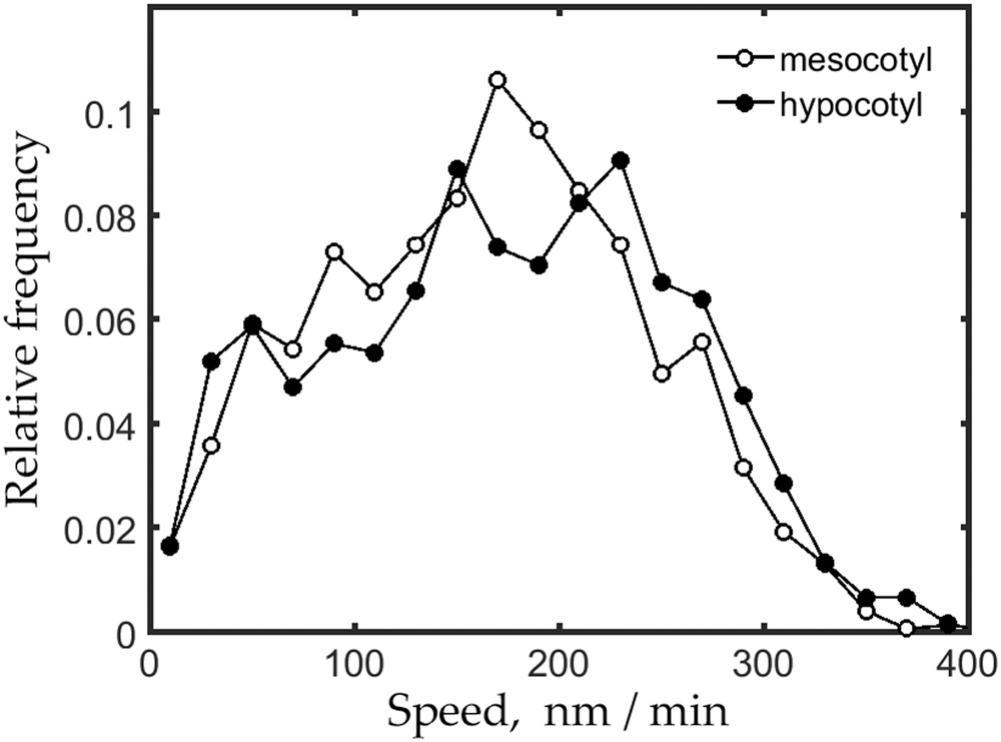
Frequency distribution for CESA speeds for *B. distachyon* mesocotyl and *A. thaliana* hypocotyl. Data are pooled from the individual sequences shown in Fig. 5. Average speed for mesocotyl equals 164 ± 78 nm min^−1^ (mean ± SD, n = 1451 particles) and for hypocotyl equals 184 ± 86 nm min^−1^ (n = 2755 particles).

To compare the species more accurately, we pooled the tracked particles from each sequence and assessed the complete distribution (Fig. 6). The distribution for *B. distachyon* appeared to be shifted to slightly lower values throughout its range. The mean CESA speed for the *B. distachyon* mesocotyl was 164 ± 78 nm min^−1^ (mean ± SD, n = 1451 particles) and for *A. thaliana* hypocotyl was 184 ± 86 nm min^−1^ (n = 2755 particles). Based on a *t*-test, there is less than one chance in a thousand that these means are the same. Nevertheless, the means differ by an amount that is numerically small while the variability between cells is large (Fig. 5), circumstances that hinder assessing what if anything the statistical difference means biologically.

**Figure 7:**
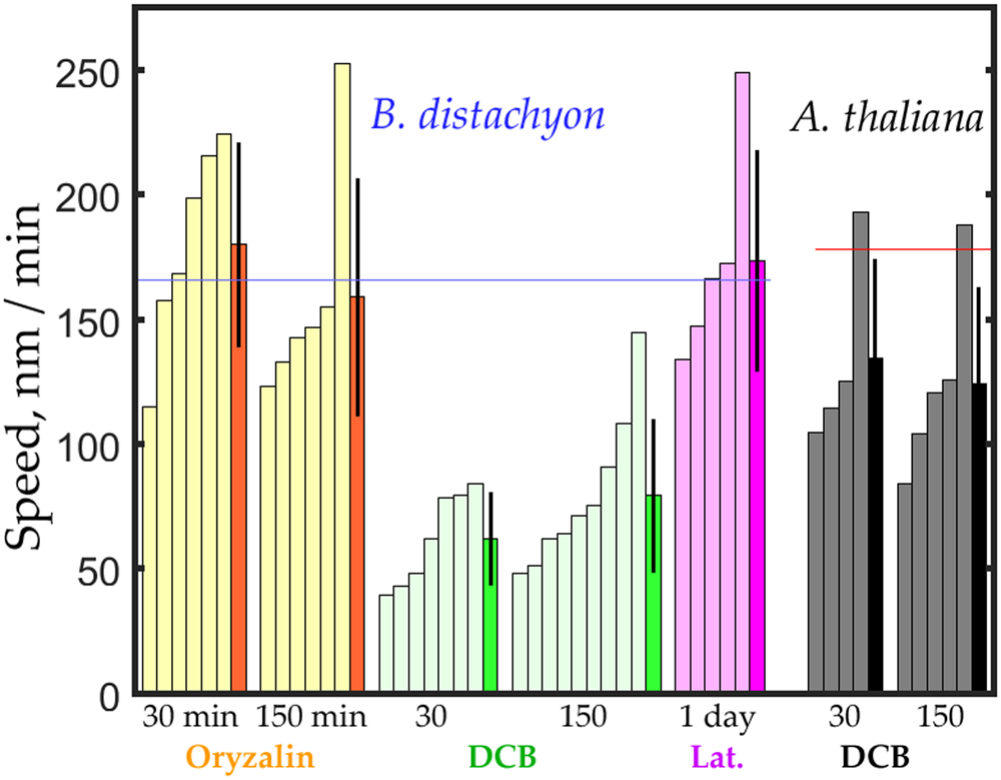
Quantification of CESA motility in response to selected inhibitors. For each treatment, lightly shaded bars plot average speed for tracked particles in a sequence. Darker bars plot mean ± SD of the average speeds shown. Horizontal lines plot the mean speed for the untreated *B. distachyon* mesocotyl (blue line) and *A. thaliana* hypocotyl (red line) from Fig. 5. Numbers beneath the bars give time of treatment (in minutes unless specified otherwise). Bars are arranged from smallest to largest for clarity. For oryzalin (30 nM), tracked particle number per sequence ranged from 26 to 323; for DCB (5 µM) (*B. distachyon*), from 22 to 207; for latrunculin (250 nM), 16 to 100; and for DCB (5 µM) (*A. thaliana*) from 12 to 294. Experiments for oryzalin were done on two occasions with two plants each; latrunculin on one occasion with three plants; DCB in *B. distachyon* on three occasions with two to four plants each; DCB in *A. thaliana* on one occasion with three plants.

Finally, we quantified CESA motility for the sequences obtained for the *B. distachyon* mesocotyl in seedlings exposed to the selected inhibitors (Fig. 7). Confirming visual inspection, the average speeds for oryzalin or latrunculin treatments were indistinguishable from those of the control. In contrast, 30 minutes of DCB was sufficient to decrease average speed by 50%, an effect that did not become larger by 150 minutes of treatment. In contrast, DCB was reported for the *A. thaliana* hypocotyl to bring CESA motility to a standstill^22^. Therefore, for comparison, we treated *A. thaliana* with DCB and observed that mean CESA speed was reduced, but to a lesser extent than in *B. distachyon*. Taken together our results demonstrate that CESA motility in growing organs is substantially conserved in these species.

## Discussion

The *B. distachyon* mesocotyl is amenable for imaging CESA motility. In the etiolated state, it is thin enough to mount for microscopy with sub-critical angle illumination and there are abundant puncta available for tracking. Similar to the *A. thaliana* hypocotyl, the mesocotyl cytoplasm streams vigorously and contains labeled Golgi bodies and vesicles, which sometimes come close to the plasma membrane. Roots also could be imaged, provided that root hairs were eliminated. Possibly because this type of microscopy is sensitive to exact geometry, samples varied considerably in the apparent density of puncta and their overall brightness. This was true for both *B. distachyon* and *A. thaliana*. Therefore while we were able to quantify the speed of the puncta reliably, we quantified neither CESA density nor brightness.

For these experiments, we expressed tagged CESA sequences from *B. distachyon* in a wild-type background; thus, we cannot guarantee that the imaged constructs are functional. Nevertheless, the tagged proteins are probably functional. First, the constructs made here for *B. distachyon* were designed to mimic constructs of *A. thaliana* CESA genes that are functional^7,15^. Second, in some of our transgenic lines, aberrant phenotypes occurred and were correlated with high expression of the transgene; in contrast, the wild-type appearance of the lines used for imaging suggests that at least gain-of-function phenotypes are absent. Finally, in *A. thaliana*, tagged CESA genes expressed in a wild-type background have a pattern of motility that is indistinguishable from that observed when the tagged CESA gene is expressed in the cognate mutant background^27,28^. Until mutants in *B. distachyon* CESA genes for the primary cell wall are isolated, studying CESA motility in a wild-type background will be a necessary expedient. To the best of our knowledge, there are no such mutants available in any grass species.

Compared to the *A. thaliana* lines expressing the transgene in a mutant background, the more or less similar fluorescence in our *B. distachyon* lines presumably reflects a more or less similar ratio of tagged to un-tagged subunits in motile complexes. Be that as it may, tagged subunits appear to be incorporated to a substantial extent and if this had caused an appreciable decrement in the rate of cellulose synthesis, then we would expect to have seen short and swollen seedlings.

We find that CESA particles in *B. distachyon* had a mean speed and a frequency distribution closely resembling that of the *A. thaliana* hypocotyl, albeit shifted to slightly lower values. Nevertheless, the mean speed we measured for CESA movement in the hypocotyl (184 nm min^−1^) is lower than usually reported for this material. From a survey of 15 papers that report CESA speeds, the average speed is 295 nm min^−1^ and the lowest value is 209 nm min^−1^ (Table 1). Our value is thus lower than previously reported.

**Table 1:** Some published values of CESA motility analyzed by kymograph.

Speed, nm/min	Reporter	Reference
207 ± 66	GFP-CESA3	39
216 ± 70	tdT-CESA6	41
231 ± 24	GFP-CESA3	45
250 ± 37	YFP-CSA6	22
257 ± 119	GFP-CESA5	27
269 ± 128^a^	GFP-CESA5	27
280	YFP-CESA6	21
290 ± 92	YFP-CESA6	42
293 ± 54	YFP-CESA6	40
324	G/YFP-CESA3/6	9
330 ± 65	YFP-CESA6	7
353	YFP-CESA6	44
353 ± 68	GFP-CESA6	43
361 ± 163^b^	GFP-CESA6	43
409 ± 173	YFP-CESA6	46
**295 ± 59** ^**c**^		

^a^Light-grown plants; all other values are for dark grown plants.

^b^Data from the paper’s supplement.

^c^Average ± SD.

For the data in Table 1, CESA speed was quantified by using kymographs. To determine whether our low rate reflects biology (e.g., growth conditions) or an analytical difference between kymographs and particle tracking, we analyzed a subset of our image sequences by using kymographs. For a total of 513 particles, the kymograph rate was 276 ± 6 nm min^−1^ (mean ± SE), a value well within the range of those in the literature but higher than the rate we found by particle tracking on the same sequences (209 ± 2 nm min^−1^, 1340 particles). Therefore, our rate appears to be low because of methodology rather than biology.

Particle tracking and kymographs sample CESA particles differently. Tracking applies an automated methodology that requires particles conform to a fixed size and minimum brightness; particles that are adjacent, cross paths, or blink substantially are likely to be excluded. On the other hand, the kymograph has subjective steps. In particular, the kymograph begins with the user drawing a line on the image superimposed on a line in the projection image representing coherent movement. In projection, the faster and longer-lived the particle, the more prominent the line, and thus users might be drawn to over-sample rapidly moving, longer-lived complexes. Even though the basis for the numerical discrepancy remains to be elucidated, we believe that this discrepancy should not detract from our conclusion that CESA particles in *B. distachyon* and *A. thaliana* move at essentially the same speeds.

As reported for *A. thaliana*, we found for *B. distachyon* that CESA speed was affected by treatment with neither a microtubule inhibitor (oryzalin) for a few hours nor with an actin inhibitor (latrunculin) for a day. Interfering with actin causes root swelling in *B. distachyon*, as it does in *A. thaliana*, although the swelling is substantially less than caused by inhibiting microtubules. Inhibiting actin has been associated with altered delivery of cellulose synthase complexes to the plasma membrane apparently through interfering with the movement of Golgi bodies^23–25,29^. Although we did not analyze the density or patchiness of CESA puncta in the presence or absence of latrunculin, qualitatively the two conditions appeared similar.

The cellulose synthesis inhibitor, DCB, inhibited CESA motility by about 50%. In *A. thaliana*, the compound was reported to stop CESA movement completely and to increase the density of CESA puncta at the plasma membrane^22^. Nevertheless, we found with particle tracking that DCB caused a reduction of CESA speed in both species, rather than a cessation. Insofar as the effects of DCB on CESA motility in *A. thaliana* have been reported apparently in only one publication, it is difficult to assess why we report a less severe effect. While methodology might again be at play, tracking apparently reports a higher speed than kymographs for DCB treatment, which is the opposite of what happens for untreated material.

Compared to DCB, the cellulose synthesis inhibitor isoxaben has been used more frequently to perturb CESA motility. However, isoxaben was developed as a herbicide for grass crops and consequently these species are relatively insensitive. As for DCB, while this compound affects *A. thaliana* and *B. distachyon* over a similar range of concentrations (Fig. 3), DCB might exert a weaker effect on cellulose synthesis in grasses compared to eudicots. In *A. thaliana*, roots exposed to a saturating dose of DCB swell to three times their initial diameter^17^; whereas, those of grasses swell to a more limited extent (Fig. 3; ref.^30^). Furthermore, while DCB is categorized as a cellulose synthesis inhibitor^31^, investigations that established this mechanism were done on eudicot species mainly. In one example for a grass (maize roots), DCB inhibited cellulose synthesis rather modestly and in addition stimulated synthesis of hemicellulose^32^. While DCB inhibits elongation similarly in *B. distachyon* and *A. thaliana*, compounds closely related to DCB are powerful inhibitors of electron transport^31^ that also inhibit growth. Even in eudicots, the molecular mechanism of DCB action against cellulose synthesis remains to be elucidated and mechanisms in grass species might be partially distinct.

Compared to each other, *B. distachyon* and *A. thaliana* have cellulose synthase complex motility that is almost indistinguishable, including the average speed of all particles measured, the variation in average speed among cells, the shape of the relative frequency distribution, and the response to DCB. The overall uniformity of cellulose synthase motility between these two species suggests that the operation of the complex is tightly constrained.

## Materials and Methods

### Seedling growth and handling

*Brachypodium distachyon* (L.) line Bd 21-3 was used, except that the cDNA used for cloning was made from Bd 21, which is the reference genome. Seeds were stripped of glumes and surface-sterilized in 70% ethanol for 2 min, in 20% household bleach and 0.1% Triton X-100 for 5 min, and then rinsed at least three times in water. Seeds were plated on half-strength Murashige-Skoog (MS) medium (pH 5.7), solidified with 0.8% Bactoagar. In some cases, plates were supplemented with 10 µM AgNO_3_, added to medium after autoclaving. After stratification at 4 °C for at least 5 days, plates for tagged CESA imaging were wrapped with aluminum foil, and placed vertically for 3 to 4 days in a growth chamber at 25 °C. For dose-response experiments, plates were exposed to continuous light (approximately 100 µmol m^−2^ s^−1^). Seeds from an *Arabidopsis thaliana* (L.) line (Columbia background) that expresses *GFP-CESA6* ^15^ were sterilized, stratified, and grown on modified Hoagland’s medium, as described by Rahman *et al*.^18^ except that plates were placed in the dark at 22 °C.

Oryzalin, DCB, and latrunculin B were dissolved in dimethyl-sulfoxide (DMSO) to create stock solutions and stored at −20 °C. For dose-response curves, agar-nutrient plates were made by adding an appropriate volume of stock solution to molten agar, with controls receiving the same volume of DMSO. Five-day-old wild-type seedlings were transferred onto the plates, the position of the root tip scored on the back of the plate and the plates returned to the growth chamber. After the indicated interval, root tip positions were scored again, the plates scanned, and elongation was measured by using ImageJ^33^ on the scanned images as the distance along the root between the two score marks. Immediately after scanning the plates, root diameter was measured through a stereo dissecting microscope either from captured images or directly by eye with an ocular micrometer. In swollen roots, diameter was measured at its apparent maximum; for others, diameter was measured at the position where root hairs become full grown.

### Construction of mEGFP-BdCESA transgenic lines

Total RNA was extracted from *B. distachyon* tissue (7-day-old whole seedlings) and reverse transcribed into cDNA with Invitrogen SuperScript III Reverse Transcriptase. Specific primers (with added attB sites) were used to amplify *CESA3* (Bradi1g54250.1) and *CESA6* (Bradi1g53207) coding sequences into pENTRp5p2 (Table 2). For tagging, we used *mEGFP* ^14^ in pENTRp1p5r. A multi-site Gateway reaction united the *mEGFP* and the *CESA* coding sequences in the plant expression vector pIPKb002^34^, in which expression of the tagged *CESA* genes is driven by a ubiquitin promoter from *Zea mays*. All constructs were confirmed by digestion with SfiI and sequencing.

**Table 2:** Primers used in this study.

*For cloning and sequencing:*
mEGFP3′seq: 5′ CTT AGC AAA GAC CCC AAC GAG A 3′
BdCESA3 (Bradi1g54250) CDS in pENTRp5p2: (BdCESA3attB5-2F) 5′ GGGG ACA ACT TTG TAT ACA AAA GTT GAC ATGGACGTCGACGCGGGTGC 3′
BdCESA3attB2-2R0 5′ GGGG AC CAC TTT GTA CAA GAA AGC TGG CTAGCAGTTGATGCCACAGG 3′
BdCESA6 (Bradi1g53207) CDS in pENTRp5p2: (BdCESA6attB5-F) 5′ GGGG ACA ACT TTG TAT ACA AAA GTT GGC ATGGAGGCGAGCGCGGGG 3′
BdCESA6attB2-R 5′ GGGG AC CAC TTT GTA CAA GAA AGC TGG TTAGTTGCAATCCAGACCACAT 3′
*For RT-PCR:*
mEGFP-F: CCCAGTCCAAGCTTAGCAAAG
BdCESA3-R: CACGGATCGCTGGGCTC
BdCESA6-R: CGCTCGTACTCGTAGCA
UBC18-F: GGAGGCACCTCAGGTCATTT
UBC18-R: ATAGCGGTCATTGTCTTGCG

Plant lines were made following an established protocol^35^. Briefly, plasmids containing *mEGFP-BdCESA3* and *mEGFP-BdCESA6* were transformed into agrobacterium line AGL1 by electroporation, and positive clones were tested with colony PCR. Transformed AGL1 lines were co-cultured with 3-month-old callus derived from embryos, and after three rounds of hygromycin selection, surviving callus was transferred onto regeneration medium. Regenerated plants were transferred to soil and propagated in the greenhouse.

For checking expression of the transgenes, we employed semi-quantitative RT-PCR, with primers shown in Table 2. The cDNA was synthesized (as above) with RNA isolated from leaves of 3-week-old plants. As a loading control, we used ubiquitin conjugating enzyme 18 (UBI18) based on its stable expression^36^.

### Imaging

At the time of imaging, *B. distachyon* seedlings were 3 to 4 days old and *A. thaliana* were 4 to 5 days old. Whole seedlings were mounted in water under a coverslip, previously cleaned for 1 min in a plasma, and sealed with a home-made mixture of Vaseline, lanolin, and paraffin (“VALAP”). For inhibitor treatment, chemicals were freshly diluted in liquid growth medium immediately before treatment. Controls received DMSO only. Three-day-old seedlings were treated with inhibitor in liquid growth medium for the indicated times. For longer treatments, two-day-old seedlings were transplanted onto plates that contained the inhibitor and incubated vertically in darkness at 25 °C. Plates containing inhibitors were made as described above for the dose-response experiments.

Imaging was performed on an Olympus inverted microscope (IX81) set up for total internal reflection microscopy. Fluorescence was collected through a 60X, 1.45 NA oil immersion objective. The mEGFP was excited at 488 nm by an argon ion laser beam passing through the exciting filter Chroma ZET488 and reflected off of a dichroic mirror (Semrock Di01-R405/488/ 561/635). Fluorescent emission was collected through the band pass emission filter (Chroma ET 525/50bp-OD8). Excitation energy at the samples was adjusted to 1.0 mW power. Images were acquired with an Andor iXon DU897E camera in EMCCD gain mode, driven by Metamorph software. Vertical drift was corrected by autofocus (Olympus IX2-ZDC2). Typical exposures were 100 or 500 msec. Frame intervals for *B. distachyon* mesocotyl were 0.6 or 1.6 sec; for the root, frames were averaged and the interval between averaged frames was 0.6, 1.6, or 1.9 sec; and for the *A. thaliana* hypocotyl were 1, 2, or 3 sec. Because the presence of the cell wall precluded the use of total internal reflection, the angle of illumination was adjusted individually for each set of images to minimize background while permitting observation of the cell membrane.

When present, image drift in the focal plane, primarily due to growth of the plant, was corrected by using the program *distribution field tracking*, implemented for MATLAB^37^. This program finds the best match of a specified image region (the region to be stabilized) in the next frame. Unlike programs that compare image regions pixel-by-pixel, this one represents each image location with a normalized histogram (or probability distribution) representing the image values in the vicinity of each pixel^37^. This allows for good matches of one image patch to another, even when there are small perturbations in the positions of image elements, since it is the distribution of nearby values that must match, rather than the exact pixel values. The distribution field tracking algorithm is able to find stable matches of an image region even when there is significant variability in its exact appearance from frame to frame.

CESA particles were tracked by using another MATLAB program^26^. Particles were identified by using a mask that selects features with a radius approximately equal to 3 pixels (the “feature size”). Particles were rejected if the calculated radius of gyration squared was greater than 6 pixels squared, or if the maximum intensity was less than several standard deviations above the background intensity for the sequence. All particles had an eccentricity of approximately 0.5, most likely limited by the TIRF optics, so eccentricity was not used as a criterion. To link particles between frames and form trajectories, the maximum displacement between successive frames was set to 2 pixels. Because GFP blinks, we permitted a particle to skip 1 frame; a trajectory was ended if a particle went dark for more than one successive frame. Additional post-processing was used to further reject trajectories with marginal particle identification due to blinking or a low signal-to-noise ratio. Specifically, a region outside of the mask was used to define a background intensity, then the integrated background was subtracted from the integrated intensity of each feature. If the result was negative, or if the localization error^38^ associated with the feature is larger than one pixel, then the feature is dropped from the trajectory. If this resulted in a particle being absent for more than one successive frame, the trajectory was omitted. All particle trajectories that lasted more than 30 seconds were used.

For each particle trajectory, speed was calculated as the vector sum of the regression coefficients of the *x* coordinate versus time and the *y* coordinate versus time.

For kymograph analysis, we used the ‘Multi-Kymograph’ plug in (http://imagej.net/Multi_ Kymograph) for Image J, as described by those authors.

### Data availability

The datasets generated for the current study are available from the corresponding author on reasonable request.

## Electronic supplementary material

Movie Captions

Movie S1

Movie S2

Movie S3

Movie S4

Movie S5

Movie S6
