# Supplementary material for: Imaging cellulose synthase motility during primary cell wall synthesis in the grass Brachypodium distachyon
Source: Sci Rep. 2017 Nov 8;7:15111. doi: 10.1038/s41598-017-14988-4 (PMC5678151; doi:10.1038/s41598-017-14988-4)
Supplement: Supplementary file 1 — Movie Captions [file 41598_2017_14988_MOESM1_ESM.pdf]

Derui Liu, Nina Zehfroosh, Brandon L. Hancock, Kevin Hines, Wenjuan Fang, Maria Kilfoil,

Erik-Learned-Miller, Karen A. Sanguinet, Lori S. Goldner, Tobias I Baskin

Imaging cellulose synthase motility during primary cell wall synthesis in the grass *Brachypodium distachyon*

Supplementary information

Movie S1

Brachypodium distachyon mesocotyl. GFP tagged CESA3 particles imaged with instrumentation for total internal reflection fluorescence (TIRF) microscopy but illuminated at a sub-critical angle. The movie contains 150 frames, taken with 0.5 sec exposure time and a 1.6 sec frame interval. Shown here at 45 times real time.

Movie S2

B. distachyon root. GFP tagged CESA3 particles imaged with instrumentation for TIRF microscopy but illuminated at a sub-critical angle. The images were acquired with 0.1 sec exposure time. The movie contains 200 frames where each frame is an average of 5 frames and the time interval between frames is 0.65 seconds. Shown here at 19.4 times real time.

Movie S3

Arabidopsis thaliana hypocotyl. GFP tagged CESA6 particles imaged with instrumentation for TIRF microscopy but illuminated at a sub-critical angle. The movie contains 500 frames, taken with 0.1 sec exposure time and a 0.13 sec interval between frames. Shown here at 4 times real time.

Movie S4

B. distachyon mesocotyl treated with 30 nM Oryzalin for 30 minutes. GFP tagged CESA3 particles imaged with instrumentation for TIRF microscopy but illuminated at a sub-critical angle. The movie contains 150 frames, taken with 0.5 sec exposure time and a 1.6 sec frame interval. Shown here at 45 times real time.

Movie S5

B. distachyon mesocotyl treated with 5 µM DCB for 150 minutes. GFP tagged CESA3 particles imaged with instrumentation for TIRF microscopy but illuminated at a sub-critical angle. The movie contains 150 frames, taken with 0.5 sec exposure time and a 1.6 sec frame interval. Shown here at 45 times real time.

Movie S6

B. distachyon mesocotyl treated with 250 nM latrunculin for 60 minutes. GFP tagged CESA3 particles imaged with instrumentation for TIRF microscopy but illuminated at a sub-critical angle. The movie contains 150 frames taken with 0.5 sec exposure time and 1.6 sec time interval. Shown in 45 times real time.
